# Cyproheptadine Treatment in Children and Adolescents with Migraine: A Retrospective Study in Japan

**DOI:** 10.3390/neurolint16060099

**Published:** 2024-10-30

**Authors:** Hideki Shimomura, Sachi Tokunaga, Eisuke Terasaki, Naoko Taniguchi, Yohei Taniguchi, Saeka Yoshitake, Yuki Terakita, Kenji Inoue, Masumi Okuda, Takeo Kato, Yasuhiro Takeshima

**Affiliations:** 1 Department of Pediatrics, Hyogo Medical University School of Medicine, Nishinomiya 663-8501, Japan; 2 Department of Pediatrics, Shiga Medical Center for Children, Moriyama 524-0022, Japan

**Keywords:** children, migraine, prophylactic treatment, cyproheptadine, comorbid conditions

## Abstract

Objective: Evidence on prophylactic drugs for pediatric migraine is limited, especially when comorbid conditions contribute to treatment resistance. This study evaluated the efficacy of cyproheptadine in children with migraine and explored the impact of comorbid neurodevelopmental disorders and orthostatic intolerance (OI). Methods: We retrospectively analyzed pediatric migraine patients treated with cyproheptadine. Efficacy was assessed based on the reduction in headache frequency, with responders defined as patients experiencing at least a 50% reduction in headache episodes. Fisher’s exact test analyzed the relationship between efficacy and comorbid conditions or treatment sequence. Multiple logistic regression was performed to identify factors associated with adverse events. Results: In total, 155 children (71 males, 84 females) aged 3–15 years were included. Comorbid neurodevelopmental disorders and OI were present in 27 (17.4%) and 22 (14.2%) patients, respectively. Efficacy was evaluated in 148 patients, with 68.9% classified as responders. Patients with comorbid conditions showed lower efficacy. Responders required a lower dose of cyproheptadine (*p* = 0.039). Multiple logistic regression identified headache frequency, cyproheptadine dose, and comorbid OI and neurodevelopmental disorders as factors influencing treatment efficacy. Conclusions: Cyproheptadine is effective in treating pediatric migraine, though patients with neurodevelopmental disorders and OI demonstrated reduced efficacy.

## 1. Introduction

Headache is one of the most common complaints among children and adolescents. Recurrent headaches significantly affect the quality of life and social functioning of children, adolescents, and their families [1,2]. Non-pharmacological treatments are recommended as the first-line treatment for migraine headaches. When non-pharmacological approaches are ineffective, prophylactic drug treatment should be considered. The efficacy of several drugs, including amitriptyline, propranolol, topiramate, and flunarizine, has been investigated for migraine prophylaxis [3,4]. A recent meta-analysis on preventive medications for pediatric migraine [5] reported that topiramate and pregabalin were associated with a reduction in headache frequency and intensity. While other drugs, including flunarizine, riboflavin, amitriptyline, and cinnarizine, also demonstrated statistically significant outcomes, further investigation is needed to substantiate these findings. No correlation was identified between the use of these medications and improvements in quality of life or the duration of migraine attacks. Open-label studies have reported clinically meaningful effects in the preventive treatment of pediatric migraine [6,7]. The lack of conclusive RCT data may be attributed to factors such as limited pediatric populations in trials, failure to account for comorbid conditions or insufficient long-term follow-up. Small sample sizes and the heterogeneity of clinical presentations further compound this challenge.

Cyproheptadine is a histamine receptor H1 antagonist and a prophylactic drug that has long been commonly used in children with migraine [8]. Although there are fewer reports on the effects of cyproheptadine on migraine compared to other currently available drugs, some studies, including RCTs, have reported its therapeutic effects in adults. It has been shown to improve the frequency, duration, and severity of migraine attacks [9,10]. Despite the long-standing clinical utilization of this medication, there is a notable paucity of published literature on the treatment of migraine in children [8]. A few studies have reported its efficacy, but the results have been inconsistent [11,12]. The administration of cyproheptadine for gastrointestinal disorders has been reported to cause somnolence and weight gain in a substantial proportion of patients [13]. Additionally, cyproheptadine can cause behavioral changes in some children, such as aggression [14].

Understanding comorbid conditions associated with migraine is important for clinicians to effectively diagnose and manage migraine in children and adolescents. Various comorbid conditions have been reported, including physical diseases such as asthma, atopic dermatitis, epilepsy, obesity, and sleep disorders, as well as psychiatric disorders like neurodevelopmental disorders, depression, anxiety, and emotional problems [15,16,17,18,19]. Children with orthostatic intolerance (OI) also frequently experience recurrent headaches [20,21,22]. OI has been identified as a condition characterized by autonomic nervous system dysfunction, which manifests as difficulty tolerating an upright posture. The symptoms associated with OI typically subside when the patient returns to a supine position [20]. OI is broadly classified into orthostatic hypotension (OH), where blood pressure decreases upon standing, and postural tachycardia syndrome (POTS), where the pulse rate increases. However, the detailed classification can vary among researchers. Although headaches have been reported in both OH and POTS, there are few studies on the effectiveness of pharmacological treatments for these headaches. Although detailed manifestations and treatment outcomes of headaches associated with each comorbid condition are scarce, clinical experience suggests that these headaches are often severe and intractable.

We hypothesized that migraine associated with comorbid conditions reduce the therapeutic efficacy of prophylactic drugs and that the presence of comorbid conditions enhances the likelihood of adverse events. This study aimed to compare the effects of cyproheptadine on patients suffering from migraine with and without comorbid conditions, including neurodevelopmental disorders and OI.

## 2. Materials and Methods

### 2.1. Patients

We retrospectively analyzed the medical records of pediatric migraine patients treated with cyproheptadine between 2016 and 2021. Migraine was diagnosed in accordance with the International Classification of Headache Disorders, 3rd Edition (ICHD-3) [23]. In the diagnosis of migraine, both migraine with aura and migraine without aura were included. Data analyzed included the patient’s age at cyproheptadine initiation, age at first headache onset, sex, comorbid conditions, daily dose of cyproheptadine, headache frequency, order of cyproheptadine administration (initial, adjunctive, or switch from other prophylactic medications), and adverse events. For comorbid conditions, we focused on neurodevelopmental disorders and OI, as these are frequently associated with comorbid headaches in children. The need for informed consent was waived due to the retrospective nature of our investigation, and the study design was approved by the Review Board of Hyogo Medical University (approval number 4135). This study was conducted in accordance with the Declaration of Helsinki and other national regulations and guidelines.

### 2.2. Evaluations

To evaluate the efficacy of treatment, we assessed the reduction in headache frequency. The mean frequency of headaches was calculated over a baseline period of one month prior to the initiation of cyproheptadine and during a therapeutic period of one month, starting three months after administration. Treatment responders were defined as those reporting a reduction of at least 50% in headache frequency. Neurodevelopmental disorders were diagnosed based on the Diagnostic and Statistical Manual of Mental Disorders, Fifth Edition (DSM-5) [24], which included attention deficit hyperactivity disorder (ADHD) and autism spectrum disorder (ASD). We defined OI as meeting two or more of the following five symptoms presenting at least once a week and persisting for at least 6 months: susceptibility to vertigo and dizziness while standing; a tendency for fainting in the standing position, which in severe cases leads to falls; nausea on taking a hot bath or on encountering unpleasant experiences; palpitation and/or dyspnea after mild exercise; and difficulty in getting out of bed. These symptoms were based on the screening symptoms checklist in the Japanese diagnostic criteria for orthostatic dysregulation (OD) [21].

### 2.3. Statistical Analysis

All statistical analyses were performed using JMP Pro 16 (SAS Institute Inc., Cary, NC, USA). Fisher’s exact test assessed the relationship between treatment efficacy and the order of administration or comorbid conditions. The correlation between comorbid conditions and age at first headache onset, as well as the correlation between cyproheptadine dose and treatment effect, and between the treatment effect and the headache frequency before treatment, were calculated using the Mann–Whitney U test. Multiple logistic regression analysis assessed the factors associated with adverse events. The odds ratio (OR), a measure of association between an exposure and an outcome, was calculated. Sex, order of administration, OI, and neurodevelopmental disorders were treated as categorical variables, while age at first headache onset, age at initiation of treatment, frequency of headache before treatment, and initial dose of cyproheptadine were treated as continuous variables. The odds ratios of all continuous variables, excluding the initial dose of cyproheptadine, indicated that the odds of the outcome of interest increased with every unit increase in the input variable. Since the change in the initial dose of cyproheptadine was small, the odds ratio represents the odds for a 0.1 unit increase in this variable. The odds ratio for categorical variables was calculated as the risk for non-responders. Statistical significance was set at *p* < 0.05.

## 3. Results

The demographic characteristics of the participants are presented in Table 1. In total, 155 participants (71 male, 84 female) aged 3–15 years (median: 10 years) were included in the analysis. Comorbid conditions of neurodevelopmental disorder and OI were observed in 27 (17.4%) and 22 (14.2%) participants, respectively. Seven participants had both comorbid conditions. All patients’ median age at headache onset was 7 years old (2–14 years old). For those without comorbid conditions, the median age at headache onset was also 7 years (2–14 years). In patients with comorbid conditions, the median age at onset was 7 years (3–13 years) for those with neurodevelopmental disorders and 10 years (5–13 years old) for those with OI. Patients with OI were significantly older at headache onset than those without comorbid conditions. (*p* = 0.0002). The initial daily cyproheptadine dose ranged from 0.04 to 0.2 mg/kg (median, 0.1 mg/kg).

Adverse events were experienced in 33 (21.3%) patients. The most prevalent adverse event was somnolence, observed in 26 patients (16.8%), followed by fatigue in three patients (1.9%). Other adverse events included dizziness, dysesthesia, aggravation of headache, and visual hallucination in one patient each. Among the patients who experienced adverse events, seven (4.5%) withdrew from the study because of these events. Of these, six patients experienced somnolence, and one experienced aggravated headache. Among the seven patients who discontinued cyproheptadine treatment, only one had comorbid neurodevelopmental disorders, and none had comorbid OI. No significant risk was detected for any of the established covariates of somnolence, which was the most observed adverse event. (Table 2). Among the three patients who reported adverse events of fatigue, none had comorbid OI or neurodevelopmental disorders.

The efficacy of cyproheptadine was analyzed in 148 patients (Table 3), excluding seven patients who withdrew because of adverse events. Overall, 68.9% of patients were classified as responders. The efficacy was 70.8% for those using cyproheptadine as the initial treatment, compared to 41.7% for those using it as adjunctive or switch therapy. Although cyproheptadine tended to be less effective as adjunctive therapy, the difference was not statistically significant (*p* = 0.097). The drugs administered prior to cyproheptadine were amitriptyline in four cases and lomerizine hydrochloride in seven cases. In one patient in each of these cases, valproate was administered concurrently. When comparing efficacy between patients with and without comorbid conditions, those with neurodevelopmental disorders and OI exhibited lower efficacy (50.0%, *p* = 0.0139 and 45.5%, *p* = 0.0079, respectively). No significant relationship was observed between the treatment effect and the headache frequency before treatment (*p* = 0.197). Regarding the association between treatment efficacy and the dose of cyproheptadine, the effective cases required a lower dose of cyproheptadine (*p* = 0.039). In the multiple logistic regression analysis (Table 4), cyproheptadine efficacy was significantly associated with the frequency of headaches before treatment, cyproheptadine dose, and neurodevelopmental disorders.

## 4. Discussion

In this study, we demonstrated the efficacy of cyproheptadine in treating children with migraine. Overall, 68.9% of patients were classified as responders, with higher efficacy observed in 76.6% of cases without comorbid conditions. Efficacy was not significantly related to age, sex, or administration order. Cyproheptadine was less effective in cases of migraine associated with comorbid neurodevelopmental disorders or OI.

While treating children and adolescents with migraine is beneficial, the evidence regarding the efficacy of prophylactic drugs remains inconclusive [3]. The effectiveness of pharmacological treatments for migraine prophylaxis in pediatric patients has been extensively researched; however, the evidence supporting their efficacy remains controversial. Most RCTs investigating the efficacy of prophylactic drugs for pediatric migraine have failed to demonstrate superiority over placebos. A key reason for this may be the high placebo response in children [3,25]. Powers et al. conducted a randomized, double-blind, placebo-controlled trial to investigate the effects of amitriptyline, topiramate, and placebo on migraine prevention in childhood and adolescence [25]. No significant differences were observed in headache frequency or disability reduction. This result is likely due to the greater placebo effect observed in the pediatric population, a recognized challenge in pediatric clinical trials. This finding is consistent with previous studies examining the efficacy of treatments for pediatric migraine [3]. A meta-analysis reported that propranolol and topiramate were more effective than placebo in short-term migraine treatment in children; however, no long-term prophylactic effects have been established [26].

Placebo effects are generally more pronounced in children than adults and are influenced by psychological and social factors [27,28]. These effects are significant in both psychiatric and non-psychiatric conditions and affect the outcomes of clinical trials. Open-label placebos and the strategic use of contextual factors show promise in leveraging these effects for therapeutic benefits. Understanding and leveraging placebo effects can lead to better-designed studies and potentially more effective treatments in pediatric populations [29]. Psychological and social factors that influence the placebo effect include chronic pain, ADHD, depression, and functional abdominal pain [27,28,29].

Nevertheless, several non-RCT studies have indicated that cyproheptadine and other treatments are clinically effective. In a study involving 192 pediatric patients, Hershey et al. investigated the effects of amitriptyline on migraine [6] and found that 84.2% of patients showed improvement. However, the study was limited by its inclusion of only patients who returned to the clinic, excluding those who did not complete the evaluation. Olfat et al. conducted a comparative study on cinnarizine and amitriptyline for childhood migraine [7], demonstrating that cinnarizine is an effective treatment, with amitriptyline showing comparable efficacy.

Cyproheptadine is a histamine receptor H1 antagonist with antihistamine, anti-serotonergic, and calcium-channel blocking properties [30,31]. The primary action of cyproheptadine in preventing migraine is through calcium channel antagonism, which is unique among prophylactic migraine drugs [31]. Cyproheptadine has been used as a prophylactic treatment for childhood migraine since the 1970s [8]. Although no RCTs have been conducted to date, only a few studies have investigated the efficacy of cyproheptadine. A retrospective study of 250 children revealed that cyproheptadine was the second most prescribed preventive drug, reducing mean headache frequency from 8.4 to 3.75 per month [12]. A recent retrospective study of 45 children reported a decrease in The Pediatric Migraine Disability Assessment (PedMIDAS) score and a responder rate of 52% for cyproheptadine, where responders were defined as those with at least a 50% reduction in headache frequency. This was compared to 90% for topiramate, 75% for propranolol, and 54% for flunarizine [11].

Despite its lower efficacy compared to other drugs, cyproheptadine is often administered to younger patients due to its mild side effects, such as sedation and increased appetite, which typically do not interfere with daily life [4,11]. In this study, we examined the efficacy of cyproheptadine in patients with and without comorbid conditions and found that the response rate was higher in patients without comorbid conditions than in previous reports. Despite the absence of a direct correlation between headache frequency prior to treatment and treatment efficacy, multiple logistic regression analyses of factors affecting the efficacy of cyproheptadine identified headache frequency before treatment, cyproheptadine dose, and the presence of comorbid neurodevelopmental disorders as significant risk factors. This indicates that patients who responded to cyproheptadine required lower doses and had a higher number of headaches before treatment. It remains unclear why lower doses of cyproheptadine might be more effective. However, it is plausible that increasing the dose in response to an initial lack of effectiveness may not yield better results. Compared with patients who used cyproheptadine as their first drug, cyproheptadine had a lower efficacy in patients who received adjunctive cyproheptadine therapy or switched to cyproheptadine therapy; however, this difference was not statistically significant. This outcome was likely attributable to the relatively small number of patients. Further investigation is required to ascertain the efficacy of cyproheptadine as an adjunctive or second-line therapy. This may help to elucidate the optimal initial pharmacological intervention.

Adverse events occurred in 26% of patients, and 4.5% discontinued cyproheptadine, consistent with previous reports [4,11]. Tekin et al. reported that 51% of pediatric migraine patients receiving cyproheptadine at doses of 2 to 4 mg/day experienced side effects. Although comprehensive data were unavailable, the observed adverse events included somnolence and increased appetite. The authors indicated that these side effects did not impact patients’ daily lives, and no patients discontinued the medication. In a prospective study of adolescents and adults, Rao et al. reported that 12% of patients experienced adverse events, such as drowsiness, sleep disturbances, weight gain, fatigue, and dry mouth, when treated with 2 mg/day of cyproheptadine [9]. While the authors reported that all adverse events were minor, they did not provide details. In the present study, the most frequently observed adverse event was somnolence. Compared to previous studies, this study demonstrated a higher incidence of adverse events, some of which resulted in the discontinuation of the drug. It was hypothesized that the higher dosage used in this study, when adjusted for body weight, may have contributed to the outcome. However, this could not be confirmed, as the detailed data from previous reports was unavailable. The initial hypothesis suggested that side effects were more likely to occur in the presence of comorbid conditions, particularly in patients with OI. However, this hypothesis was not supported by the evidence. none of the patients who discontinued treatment due to adverse events had comorbid OI, and only one patient had a comorbid neurodevelopmental disorder. Additionally, multivariate analysis did not identify any risk factors associated with the appearance of somnolence. Thus, predicting the appearance of adverse events was challenging due to the absence of identifiable risk factors. These results suggest that cyproheptadine is highly effective against migraine in patients without comorbid conditions, and the occurrence of adverse events was deemed acceptable and unrelated to comorbid conditions.

Non-pharmacological treatments should be initiated prior to the administration of drug therapy [32,33]. These non-pharmacological interventions include dietary modifications, nutraceuticals, neuromodulation, and cognitive-behavioral therapy (CBT). Among these, CBT is an effective intervention for pediatric migraine, with strong evidence supporting its efficacy. Furthermore, research indicates that the effectiveness of CBT is enhanced when combined with pharmacological treatments [34,35]. Although some patients in the current study were undergoing non-pharmacological therapies, including CBT, examining these interventions in sufficient detail was not feasible due to the retrospective nature of this study, which is a limitation.

Children and adolescents with neurodevelopmental disorders often experience recurrent headaches [36,37]. A meta-analysis examining the association between primary headaches and ADHD revealed a significant association between ADHD and migraine [38]. Recent studies have reported that comorbid migraine was present in 28.4% of children with ASD [37] and in 42.7% of adults with ASD [39]. The exact underlying reasons for the association between migraine, ADHD, and ASD remain unclear; however, several explanations have been proposed. For ADHD, neurotransmitters such as dopamine, noradrenaline, and gamma-aminobutyric acid have been suggested to play a role. In the case of ASD, sensory hyper-reactivity has been highlighted as a potential factor contributing to the association [37,40]. Of note, headaches may also present as a side effect of medications used to treat ADHD, such as methylphenidate, atomoxetine, and guanfacine [41]. The present study was unable to gather information on the medications used by the patients, which represents a limitation of this study. To the best of our knowledge, there are few reports on the efficacy of treatments for migraine coexisting with neurodevelopmental disorders. Patients with neurodevelopmental disorders often have difficulty adapting to treatment, and their unique characteristics can complicate migraine management [42]. We believe that this could explain why cyproheptadine was less effective in patients with coexisting neurodevelopmental disorders.

Children with OI often complain of headaches; however, these headaches are not classified as secondary headaches in ICHD-3 [23]. Therefore, OI is categorized as a comorbid condition of primary headache. The concept of OI varies depending on the reports and country [20,21,43,44,45]. Representative terms include orthostatic hypotension (OH), OD, and postural orthostatic tachycardia syndrome (POTS). Some classifications include OH and POTS under OI [20,44], while others group them under OD [21]. The diagnosis of OH is typically confirmed by observing a decrease in blood pressure in response to orthostatic maneuvers. The extent of the blood pressure reduction varies across studies, with thresholds as low as a 20 mmHg decrease or a drop of 15% or more commonly reported. The standing test can be performed in two ways: either mechanically or actively. When performed mechanically, it is referred to as the head-up tilt test, while the active version is known as the active stand test or Schellong test. Although the Schellong test was performed in all cases, the orthostatic test could not be incorporated into the diagnostic criteria because not all patients in the study had undergone this test at our facility. Consequently, a detailed diagnosis based on blood pressure and pulse rate could not be established, which represents a limitation of the study. Regardless of these detailed classifications, comorbid headaches often occur. Tanaka et al. reported that 68% of children with OI, aged 10 to 16 years, experienced headaches [46], while Boris et al. found that over 90% of patients with POTS, with a median age of 13.1 years, reported headaches [47]. In the present study, we found that children with migraine and comorbid conditions of OI were older at migraine onset than those without comorbid conditions. Although detailed headache manifestations and diagnoses of headache comorbid with OI have not been reported, older age at onset was identified as a characteristic feature of migraine associated with OI. In contrast, although reports on comorbid OI in migraine patients are limited, a population-based study in adults found that 46% of patients with migraine experienced syncope, and 32% were diagnosed with OI [48]. It has been reported that OI and headaches can coexist; however, it remains unclear whether OI directly causes headaches. Among the limited reports on this topic, Go et al. suggested that orthostatic maneuvers induce headaches due to reduced cerebral blood flow [49]. However, these headaches resolve within a shorter period and do not meet the diagnostic criteria for a primary headache disorder. The lack of standardization in diagnostic criteria for OI, OH, and POTS presents a significant barrier to further research. This inconsistency is also why these conditions are not listed as causes of secondary headaches in the ICHD, as previously stated.

In our study, since not all patients were diagnosed using the same criteria for OI, they were categorized based on their OI symptoms. The results showed that 14.2% of patients with migraine had comorbid OI, but we could not assess the relationship with a detailed diagnosis. Cyproheptadine was less effective for headaches coexisting with OI, including those diagnosed using the established diagnostic criteria. In contrast, OI was not a significant risk factor for non-response in the multivariate logistic regression analysis. The findings indicate that other factors may increase the risk of invalidity in patients with OI; however, these were not identified in this study. To the best of our knowledge, no such reports have been published. Further research is required to confirm whether comorbid OI symptoms affect the prognosis of migraine.

The authors acknowledge several limitations in the present study. First, because this was a retrospective study, treatment was not performed based on a strict protocol. Therefore, adherence to the appropriate dose of cyproheptadine could not be confirmed. Second, the efficacy of cyproheptadine was assessed solely based on headache frequency. Utilizing additional indicators, such as headache severity (assessed by a visual analog scale or numerical rating scale) or disability (measured by the PedMIDAS or Headache Impact Test), would have provided a more comprehensive evaluation of the drug’s impact on daily life. Third, because the diagnosis of OI as a comorbid condition was based on clinical symptoms alone, determining the precise pathophysiology proved challenging. Future studies should consistently implement established orthostatic tests. Finally, other covariates may have influenced cyproheptadine efficacy. Further prospective studies are needed to establish an appropriate treatment protocol for cyproheptadine in pediatric migraine.

## 5. Conclusions

In conclusion, we demonstrated that cyproheptadine is effective in pediatric patients with migraine, particularly in those without comorbid conditions such as OI symptoms and neurodevelopmental disorders. Even in the presence of comorbid conditions, severe adverse events were uncommon, thereby establishing the drug as a safe and efficacious treatment option for migraine in children.

## Figures and Tables

**Table 1:** Baseline and demographic characteristics.

Patients enrolled, n	155
Median age, years (range)	10y0m (3y9m–15y8m)
Sex, n (%)	
Male	71 (45.8)
Female	84 (54.2)
Comorbid condition	
Neurodevelopmental disorder, n (%)	27 (17.4)
Orthostatic intolerance, n (%)	22 (14.2)
Age of headache onset, median years (range)	7 (2–14)
Without a comorbid condition	7 (2–14)
With neurodevelopmental disorder	7 (3–13)
With orthostatic intolerance	10 (5–13)
Frequency of headache per month, median (range)	20 (1–31)
Initial daily dose of cyproheptadine (mg/kg), median (range)	0.1 (0.04–0.2)

**Table 2:** Multivariate logistic regression analysis of factors that can affect somnolence.

Variable	OR	95%CI	*p*-Value
Age at which administration was initiated	1.02	(1.00, 1.04)	0.070
Dose of cyproheptadine (OR per 0.1 mg/kg)	0.50	(0.04, 3.95)	0.540
Sex	0.93	(0.37, 2.26)	0.865
Neurodevelopmental disorder	0.80	(0.23, 2.71)	0.715
Orthostatic intolerance	1.11	(0.31, 3.93)	0.255

**Table 3:** Clinical variables related to the efficacy of cyproheptadine.

Variable		Cases, n	50% Responder, n (%)	
Total		148	102 (68.9)	
	Initial therapy	137	97 (70.8)	
	Adjunctive or switch therapy	11	5 (41.7)	*p* = 0.097
Comorbid condition				
	Without comorbid condition	107	82 (76.6)	reference
	Neurodevelopmental disorder	26	13 (50.0)	*p* = 0.0139
	Orthostatic intolerance	22	10 (45.5)	*p* = 0.0079

**Table 4:** Multivariate logistic regression analysis of factors that can affect the efficacy of prophylactic drug treatment.

Variable	OR	95%CI	*p* Value
Age of first headache onset	0.99	(0.98, 1.01)	0.787
Age at which administration was initiated	0.97	(0.80, 1.18)	0.520
Frequency of headache before treatment	1.05	(1.00, 1.09)	0.036
Sex	1.04	(0.46, 2.34)	0.930
Dose of cyproheptadine (OR per 0.1 mg/kg)	0.12	(0.02, 0.75)	0.027
Order of administration	2.21	(0.55, 8.90)	0.263
Orthostatic intolerance	2.55	(0.86, 7.59)	0.093
Neurodevelopmental disorder	3.17	(1.19, 8.42)	0.021

## Data Availability

The original contributions presented in the study are included in this article; further inquiries can be directed to the corresponding author.
